# Mental Health Outcomes among Electricians and Plumbers in Ontario, Canada: Analysis of Burnout and Work-Related Factors

**DOI:** 10.3390/bs12120505

**Published:** 2022-12-12

**Authors:** Ali Bani-Fatemi, Marcos Sanches, Aaron S. Howe, Joyce Lo, Sharan Jaswal, Vijay Kumar Chattu, Behdin Nowrouzi-Kia

**Affiliations:** 1 ReSTORE Lab, Department of Occupational Science and Occupational Therapy, University of Toronto, Toronto, ON M5G 1V7, Canada; 2 Centre for Addiction and Mental Health, Toronto, ON M6J 1H4, Canada; 3 Department of Clinical and Counselling Psychology, Columbia University, New York, NY 10027, USA; 4 Center for Transdisciplinary Research, Saveetha Dental College, Saveetha Institute of Medical and Technical Sciences, Saveetha University, Chennai 600077, India; 5 Department of Community Medicine, Faculty of Medicine, Datta Meghe Institute of Medical Sciences, Wardha 442107, India

**Keywords:** burnout, apprenticeship, electricians, plumbers, mental health, work-related factors, gender, evaluation

## Abstract

(i) Background: Working in the electrical and plumbing sectors is physically demanding, and the incidence of physical injury and work disability is high. This study aimed to assess the mental health and well-being of skilled trades workers working in the electrical and plumbing sectors; (ii) Methods: Forty participants completed an online survey assessing burnout, work-related factors, and mental health issues. Data were analyzed to determine the association between demographics, the availability, and importance of work-related factors, and burnout using a two-sample Mann–Whitney U test; (iii) Results: Our findings showed that among the work-related factors, workplace safety, family commitments, income and benefits, and full-time employment opportunities might be crucial factors to keep study participants working at their current position. Financial support for external training, which was found to be the most important factor in preventing colleague-related burnout, was available to the satisfaction of approximately 50% of the participants; (iv) Conclusion: Work-related factors such as workplace safety and the availability and support for external training may be protective against all types of burnout among this population. Future studies may consider a larger sample size with a more diverse group of participants and perform an intersectional analysis to incorporate minority identities in the analyses.

## 1. Introduction

Work provides financial security [1] and access to resources [2] for people and their families; however, it can also make workers ill. The harm caused by work-related diseases considerably outweighs the harm caused by workplace injuries. A report by Safe Work Australia (2012) [3] showed that approximately 250 workers die from an injury at work each year, while over 2000 workers die from a work-related disease. According to the Construction Industry Rehabilitation Plan, approximately 83% of construction workers have experienced moderate to severe mental health issues [4]. Individuals who work in the construction industry have a high risk of mental illness, as reflected by an increased incidence of depression, anxiety, suicidal ideation, and suicide [5,6,7]. A study by Jacobsen et al. (2013) [8] surveyed workers at a construction site and showed that 9 out of 10 survey respondents reported having mental health concerns. Stocks et al. (2010) [9] found that post-traumatic stress disorder was quite high among construction workers due to workplace hazards. Internationally, suicide rates within the construction industry are disproportionately high, as males working in the construction section are among the highest occupational risk groups for suicide [10]. Evidence suggests that rates of suicide in younger and less-skilled male construction workers are double compared to other young males. The high segregation of males within the skilled trades provides some evidence as to why the rates of suicide are higher among men than females [11]. Studies [12,13,14,15,16,17,18,19,20,21,22,23] have also investigated the factors that can compromise electrical workers’ mental health and have suggested that living in danger (such as lethal electrical shock, the danger of electrical smolders, exposure to lead, and fire and explosions) has a significant impact [19]. A study by Sauter and colleagues [18] on the effect of privatization on electrical workers’ mental health found insomnia and nervousness in electrical workers were caused by tension at work and concluded that workers’ diseases were associated with how the work was organized and performed. In addition, the physical consequences of electrical accidents can have long-term effects on health and workability, including long-term emotional and cognitive consequences [20].

Mental health issues have high economic costs to societies and individuals, prevalent in the heavy labor industries, including the electrical and plumbing sectors. Approximately 400,000 workdays are lost annually in the United Kingdom due to mental health problems, mostly among construction workers. The number of suicides reported between 2011 and 2015 in the United Kingdom was 1419 cases, which was 3.7 times the UK national average [5]. The construction industry in British Columbia, Canada, reports suicide as the second largest cause of death within this population; the highest reports are men aged 40–59 years [24]. Moreover, in Australia, the rate of completed suicide among construction workers is 2 times the Australian national average [25,26]. The literature and work examining this topic are quite limited in Canada. Thus, this study will be exploratory and contribute to the lack of literature.

It is well-documented that psychosocial stressors such as “mobbing” related to the work environment can increase the risk of mental illness. Other factors associated with this include substance abuse, relationship issues, job security, financial strain, the contribution of masculine norms in the workplace and culture (e.g., self-reliance and suppressing emotion), and injury rates. It is important to monitor the impact of psychosocial risk factors on workers’ health to better understand their effects on their mental health and well-being, which in turn helps to reduce workplace injuries, prevent disabilities, and increase productivity [27]. Moreover, occupational stress significantly affects the workability of electricians [28]. Kulić and colleagues (2019) [28] showed that workability was poor at 11.5%, moderate at 25.0%, good at 26.9%, and excellent at 36.5% of workers in the electrical sector. They found higher Copenhagen Burnout Inventory scores in electricians compared to the control group, while the work ability index score was significantly higher in electricians. The results of their study showed that factors such as work schedule (e.g., night, shift work, working overtime), job performance pressures (e.g., the time limit for individual tasks, time limits pressure for tasks performance, work over-load), inadequate workspace) and safety (e.g., risks and hazards, fear of injuries), and unexpected daily circumstances were the most frequent and strongest stressors in the electricians’ workplace.

The objective of this study was to assess the mental health and well-being of employers working in the electricity and plumbing sectors who were part of the Employer Engagement Project (EEP) available to Ontario Electrical League (OEL) members in the province of Ontario, Canada. Specifically, to investigate and report the evidence of an association between mental health conditions, measured by burnout and availability of work-related factors.

## 2. Materials and Methods

### 2.1. Setting

The Ontario Electrical League (OEL) is a non-profit members-based organization representing Ontario’s electrical industry [29]. Their goal is to promote, strengthen, and represent the electrical industry in Ontario as they bring current issues in the industry (e.g., labor laws) to the attention of various government authorities through chapter meetings, conferences, seminars, trade shows, training programs, and government relations initiatives. The OEL has built a mentorship model developed through the 2018–2019 EEP to support employers in apprentice training and creating apprenticeships for the tradespeople of tomorrow.

Electrician apprenticeship is a long process (approx. five years) and employers need help at every step to create these training opportunities and ensure Ontarians have a sustainable skilled trades workforce. Recent efforts to modernize the trade, including changing previously restrictive and confusing ratio rules governing the number of licensed electricians and plumbers required for each apprentice to 1:1, are starting to remove roadblocks for employers. The 1:1 ratio has created more apprenticeship opportunities, along with a need to support employers who have never hired and trained apprentices or have not done so in many years.

This project gives electrical and plumbing employers the required tools and support to train apprentices and thereby be more effectively engaged in developing and delivering a solution to close the skills gap in these trades. To evaluate the EEP project, the OEL partnered with the University of Toronto to perform qualitative and quantitative analyses of the supports used for employers and apprentices to identify future supports and evaluate the effectiveness of the current supports. Furthermore, we evaluated the mental health and well-being of employers in the electricity and plumbing sectors participating in the EEP. The study protocol was approved by the Institutional Research Ethics Board of the University of Toronto.

### 2.2. Study Design and Participants

This study was part of a sequential, explanatory mixed-method research design to enable a rich understanding of the OEL mentorship program and its context and to explore ideas and outcomes relevant to the needs of knowledge users. Qualitative methods were used to evaluate the efficacy of the OEL’s employer mentorship program through the perspectives of small-to-medium-sized employers using evidence-based approach. Two focus group discussions, 60–70 min in duration, were conducted virtually with 11 small-to-medium-sized employers. Focus group audio transcripts were recorded and transcribed for thematic analysis using NVivo 12 (version 12.7.0) [30].

The present study examined the mental health outcomes among electricians and plumbers (small-to-medium electrical and/or plumbing contractors with less than 50 employees/apprentices) in Ontario. This 12-month study commenced on 1 April 2021. The OEL enhanced its pilot project approach to include new pre-screening technologies and methods from one of its external service providers. After this initial contact, in-house staff at the OEL conducted more in-depth interviews to assess the employers’ readiness for entering into the project. Once the OEL established their target employers, they referred them to the University of Toronto to evaluate their mental health and workplace stressors. Study participants (*n* = 40) were recruited among OEL members involved in EEP between April 2021 and March 2022. Inclusion criteria for the study included: small-to-medium-sized employers aged 18 to 70 years, enrollment in the EEP, verbal proficiency in English, and capacity to consent to research. The resources provided by the OEL in this mentorship program include employer outreach, mentoring, training, and hiring tools. Employer outreach was achieved by contacting employers who were not currently participating in apprentice training and by educating the employer on effective business practices and the advantages of training apprentices.

### 2.3. Data Collection

Participants were invited by the OEL to take part in our online survey using an email script that was created at the University of Toronto. Informed consent was obtained from all participants. The survey measures were developed by the principal investigator (B.N.-K.) [31] using validated questionnaires and were administered by trained research staff. The survey questionnaire was a 38-item questionnaire that helped in collecting demographic data, including gender and sex, age, ethnicity, marital status, educational attainment, years of experience working as an electrician or a plumber, the number of years working, hours of work per day and in a week, overtime hours worked, income, and travel time required for work.

The questionnaire also included an inventory of factors experienced as occupational stressors and burnout based on the Copenhagen Burnout Inventory (CBI) [32] and the National Institute for Occupational Safety and Health Generic Job Stress Questionnaire (NIOSH Generic Job Stress Questionnaire) [33]. The CBI is a questionnaire with three subdivisions: personal burnout, work-related burnout, and client-related burnout. Personal burnout is the degree of physical and psychological tiredness and exhaustion suffered by a worker. Work-related burnout is the degree of physical and psychological fatigue and exhaustion perceived by the person as related to their work, and client-related burnout is the degree of physical and psychological fatigue and exhaustion that the person perceives as related to their work with clients.

The CBI is a valid, reliable, and normed measure to assess burnout levels in different work sectors and has been used in both clinical practice and research as being short-lived, simple, and openly accessible [34]. In several studies, the measure showed good psychometric properties (both in validity and reliability) for measuring occupational burnout [35,36,37,38,39]. The NIOSH Generic Job Stress Questionnaire measures the most accepted contributors to occupational stress: physical environment, role conflict, level of control, administrative and co-worker support, workload, and skill demand [33]. This questionnaire is utilized worldwide for the collection of data and assessment of occupational stress research. It comprises concepts related to stressors that would be anticipated to take the lead to some form of occupational strain in the worker, as well as factors that may affect the way the worker responds to those stressors. 

### 2.4. Data Analysis

The quantitative data were analyzed in IBM SPSS Statistics for Windows (Version 25.0.) [40]. Descriptive analyses were conducted using means, standard deviations, and percentages. To investigate the association between the availability of work-related factors (e.g., safety, an opportunity for career advancement, workload allocation, etc.) and burnout scores, non-parametric Mann–Whitney U tests were used; however, the interpretation was focused on the effect sizes and their overall patterns due to small sample size and low statistical power for significance testing. Due to the expected high rate of false negatives, we used alpha = 0.10). First, the responses regarding satisfaction with the availability of work-related factors were grouped into two categories considered meaningful: (i) satisfied and (ii) dissatisfied with the availability of the factors. In particular, as a measure of association, we analyzed the differences in average burnout scores between those dissatisfied and those satisfied with the availability of work-related factors. These differences were called “effect size,” and larger positive differences (higher burnout in the dissatisfied group) were interpreted as greater evidence in favor of the factor being important to explain burnout. Positive effect sizes indicate higher average burnout among individuals dissatisfied with the factor than those satisfied with it. The proportion of individuals satisfied with the availability of a factor was interpreted as the performance of the sector on that factor (for example, a low proportion of individuals satisfied with workload allocation relative to the satisfaction with other items indicates the low performance of the sector on how workload is allocated). The association and performance were set together as we tried to assign priority for the sector to items that are associated with burnout but with relatively low performance (also meaning that they can be improved).

## 3. Results

### 3.1. Demographic Characteristics of the Study Participants

Table 1 provides a summary of the socio-demographic characteristics of the study participants. Ninety-five percent of the participants were male, with an average age of 47.97 ± 15.62 years. The findings showed that over 87% of the study participants were born and/or raised in Ontario, and more than 97% of them (*n* = 39) got their training in Ontario. Most participants were employed in the electrical sector (*n* = 37), while three were employed in the plumbing sector. There was no association between demographic characteristics and burnout scores in the study participants.

### 3.2. Importance of Work-Related Factors

**Figure 1:**
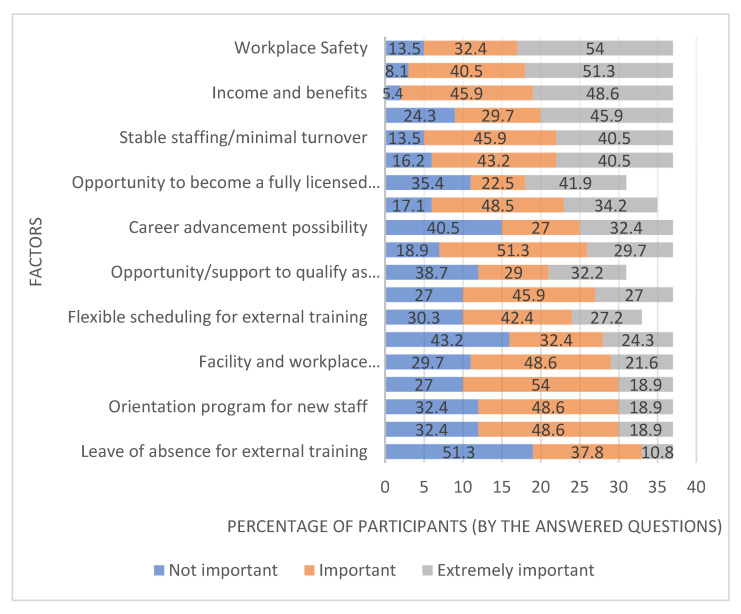
Importance of the factors that keep participants working at their current position in their current workplace.

Workplace Safety (54.0%), family commitments (51.3%), income and benefits (48.6%), and full-time employment opportunity (45.9%) were found to be the most crucial factors to keep study participants working as their current position in their workplace. Conversely, leave of absence for external training (51.3%), financial support for external training (43.2%), and career advancement possibility (40.5%) were described as the least important factors by the study participants (Figure 1).

### 3.3. Availability and Satisfaction with the Work-Related Factors in the Current Workplace

**Figure 2:**
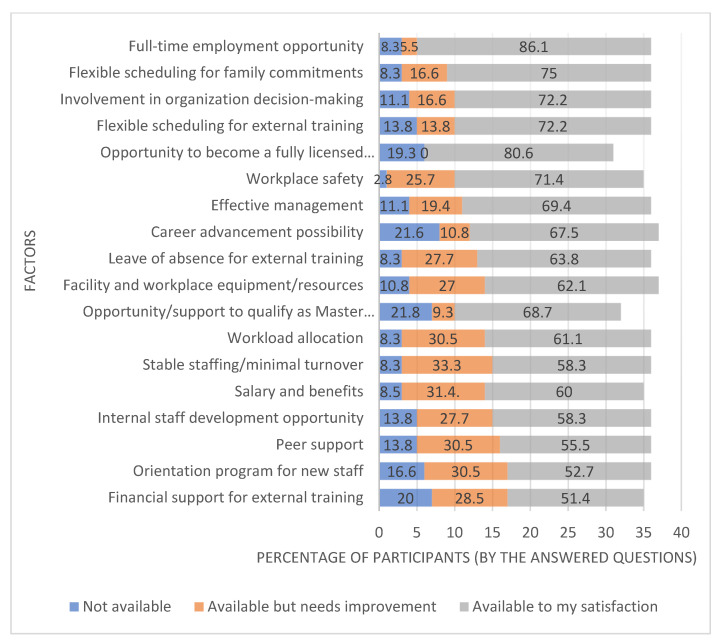
Availability and satisfaction with the following factors in the current workplace.

Full-time employment opportunity (86.1%), the opportunity for apprentices to become fully licensed electricians (80.6%), flexible scheduling for family commitments (75%), flexible scheduling for external training (72.2%), and involvement in organization decision-making (72.2%) were the most common factors that participants believed are available to their satisfaction in their current workplace. However, 21.6% of the participants believed that “career advancement possibility” was not available in their workplace (Figure 2) (refer to Table S1 in Supplementary Materials). 

### 3.4. Burnout

Table 2 shows the level of burnout in the study participants that were analyzed using the CBI (refer to Table S1 in Supplementary Materials). Our findings showed that only a few participants experienced a moderate level of burnout (average score of 50 to 74 according to CBI), including personal burnout (*n* = 7), work-related burnout (*n* = 2), and colleague-related burnout (*n* = 2). However, none of the participants reported a severe level of burnout. In the Copenhagen Burnout Inventory, scores of 50 to 74 are deemed to be ‘moderate burnout’, while scores between 75 and 99 are deemed high burnout, and a score of 100 is deemed severe burnout [33].

### 3.5. Association between Work-Related Factors and Burnout

**Figure 3:**
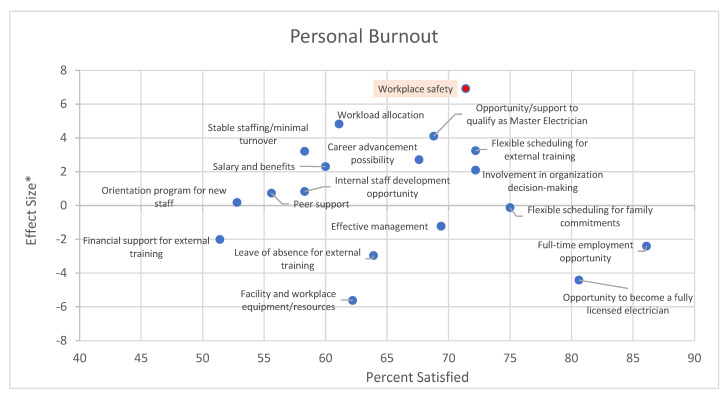
Impact of availability of work-related factors on personal burnout. * Effect sizes is the difference in the average burnout score among those satisfied and those dissatisfied with the factor. Thus, positive effect sizes indicate higher burnout among those dissatisfied with the factor. The proportion satisfied is a measure of performance that indicates the proportion of the sample that stated to be satisfied or very satisfied with the availability of the factor. The red dot indicates statistical significance at an alpha level of 0.10 when comparing the average burnout score among respondents satisfied and dissatisfied with the factor.

**Figure 4:**
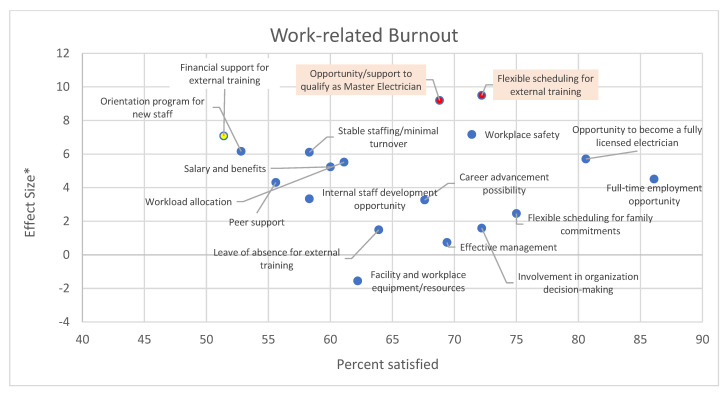
Impact of availability of work-related factors on work-related burnout. * Effect sizes is the difference in the average burnout score among those satisfied and those dissatisfied with the factor. Thus, positive effect sizes indicate higher burnout among those dissatisfied with the factor. The proportion satisfied is a measure of performance that indicates the proportion of the sample that stated to be satisfied or very satisfied with the availability of the factor. The red dots indicate statistical significance at an alpha level of 0.10 when comparing the average burnout score among respondents satisfied and dissatisfied with the factor.

**Figure 5:**
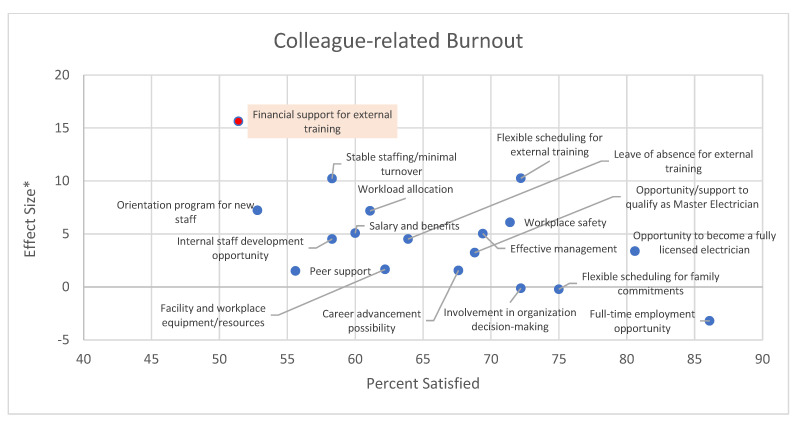
Impact of availability of work-related factors on colleague-related burnout. * Effect sizes is the difference in the average burnout score among those satisfied and those dissatisfied with the factor. Thus, positive effect sizes indicate higher burnout among those dissatisfied with the factor. The proportion satisfied is a measure of performance that indicates the proportion of the sample that stated to be satisfied or very satisfied with the availability of the factor. The red dot indicates statistical significance at an alpha level of 0.10 when comparing the average burnout score among respondents satisfied and dissatisfied with the factor.

Figure 3, Figure 4 and Figure 5 summarize the association between work-related factors and burnout in the study participants. Although we flagged the association(s) that were significant at an alpha level of 0.10 in the graphs, we focused on a more qualitative interpretation. Data on the top of the graph shows work-related factors for which dissatisfaction is associated with higher levels of burnout. Data on the left are the factors with the lowest satisfaction with their availability. Data on the top-left are associated with higher dissatisfaction associated with higher burnout scores.

Figure 3 shows factors such as workplace safety, workload allocation, opportunity/support to qualify as a master electrician, turnover, career advancements, and salary/benefits that are factors in which performance could be improved and for which dissatisfaction is associated with higher levels of burnout.

Figure 4 indicates that dissatisfaction with work-related factors is more associated with work-related burnout than personal burnout, as the effect sizes are mostly positive and larger. Financial support for external training, orientation programs for new staff, peer support, minimal turnover, salary and benefits, workload allocation, and development opportunities were among the factors that could be prioritized with a high relative effect size and satisfaction rate of approximately 60% or lower.

Figure 5 shows a similar scenario for the factors such as financial support for external training, workload allocation, minimal turnover, and orientation for new staff with larger effect sizes and an approximate satisfaction rate of 60% or lower.

## 4. Discussion

We examined the mental health outcomes among the study participants and recruitment and retention factors associated with the EEP mentorship program offered by OEL [30]. Survey data collected from 40 participants were analyzed quantitatively. The importance and availability of work-related factors, as well as the level of burnout in the study participants, were the targeted outcomes examined by the survey to assess the participants’ mental health.

Although few studies have described the impact of work-related factors on the mental health and well-being of individuals working in skilled trades, such as electricians and plumbers, the literature on the mental health in this population is scant. A sample of 38 males and two females was recruited for this study. The collected data from the survey showed that workplace safety was the most important work-related factor that kept participants in their job position at their current workplace. Prior qualitative research by our team revealed that participants appreciated the value of the OEL mentorship program through ongoing praise of the continued educational support, employer management expertise, hiring resources, and apprentice onboarding tools despite identified industry gaps in mental health stigma and accessibility [30]. In the skilled trades, there is a dearth of information regarding workplace mental health outcomes, including burnout. Given the significant labor shortage in the industry, our preliminary evidence demonstrates the importance of creating and promoting a culture of occupational health and safety in the workplace. Occupational health and safety officers (e.g., occupational therapists and occupational medicine physicians) must promote occupational health and safety programs that focus on education, mental health first aid training, recognition, and respect. This training will, in turn, help in reducing burnout and mitigating deleterious mental health outcomes. According to the survey data, only a few individuals in our sample experienced moderate burnout, primarily personal burnout. It may be caused by the small sample size and/or the nature of the sample assessed in this study.

The construction industry, including the electrical and plumbing sectors, had the second-highest rate of heavy alcohol and drug use among employees from 2008 to 2012 and had the fifth-highest rate of illicit drug use, according to a 2015 analysis of a national survey [13]. Studies have found the contribution of psychosocial factors and alcohol use in reducing electrical workers’ performance [41] and their prediction of depressive symptoms [16]. A study by de Souza et al. (2010) [22] identified that the prevalence of common mental disorders was associated with psychosocial aspects of the electricians’ workplace, especially in those with high-strain jobs as well as electrical workers with high psychological demand and low social support [22]. Similar to electricians, individuals working in the plumbing industry may also be at a higher risk of mental health problems. The reasons why plumbers are at a higher risk of poor mental health include but are not limited to long hours of working, fewer holidays and/or breaks because of the nature of this job, isolation in the work environment, and stresses related to higher rates of self-employment in this job and stress that are caused by high-pressure projects [21]. Compared with other industries, construction workers, including electricians and plumbers, suffer from high levels of burnout, work–life conflict, and early retirement due to injury [42]. Construction workers also experience higher rates of mental distress than the general male population [8,43], and the construction industry continues to have one of the highest rates of suicide [44].

Burnout is a response to chronic interpersonal stressors specified by emotional exhaustion, depersonalization, and reduced personal performance [45]. The Copenhagen Burnout Inventory was used to determine the burnout scores of the participants. The core of burnout in this tool is physical and psychological fatigue and exhaustion. Schaufeli and Greenglass defined burnout as a state of physical, emotional, and mental exhaustion that is an outcome of long-term involvement in work situations that are emotionally demanding [46]. However, burnout is not only fatigue or exhaustion and is attributed to other domains in the person’s life, including work and colleagues [32]. By collecting the personal burnout in the survey, we could compare participants’ burnout scores regardless of their occupational status. This scale is sensitive at the negative end and includes simple questions like “How often do you feel tired?”.

On the other hand, the work-related burnout questions focused on the participant’s own attribution of symptoms to their work. Therefore, we did not assess causality in the scientific sense of the term as individuals can ascribe their symptoms to their work without good scientific reason and vice versa [32]. By comparing personal burnout and work-related burnout, we could recognize the reason for burnout in the study participants if it was related to non-work factors such as health problems or family demands, or work factors. Finally, using a colleague-related burnout questionnaire, we assessed the degree of physical and psychological fatigue and exhaustion in the participants related to their colleagues at work.

Few studies have investigated burnout in electricians and plumbers. A study by Bakare and colleagues [47] investigated the level of burnout among electrical and building technology undergraduate students in Nigeria. Their findings showed that the levels of burnout were high in this population. Contrary to the findings of this study, the results of our study on the employers and employees showed that only a few participants had moderate burnout, and no participants were identified with high or severe burnout. The lower number of apprentices/employees (*n* = 5) compared to the number of employers (*n* = 35) who participated in this study may explain this contradiction. There is strong evidence of the mediation effects of the emotional exhaustion dimension of burnout on the relationship between job stress and workers’ intentions to quit [48]. The results in this study confirm the findings in the literature as 94% of the participants with a low average score of burnout had a high intention to stay in their current position in their current work in the next five years. This study also revealed that workplace safety and flexible scheduling for external training were the most protective factors against personal and work-related burnout, and more than 70% of the study participants indicated that these factors are available to their satisfaction in their workplace. However, financial support for external training, which was found to be the most important factor in preventing colleague-related burnout, was available to the satisfaction of approximately 50% of the participants. Since the rate of satisfaction with the availability of financial support for external training is low, the availability of this factor to the satisfaction of employers/employees in their workplace may be taken into consideration. The results of this study may be replicated by further studies with a larger sample size and participants from different ethnic groups and gender that includes other marginalized identities to help the policymakers to improve the workplace environment with increased availability of those work-related factors that protect both employers and employees from burnout.

According to Statistics Canada [49], approximately 60% of employment opportunities in 2017 were gained by immigrants. The report also shows that almost 80% of Canada’s indigenous population is under 55 years. Moreover, nearly 48% of the Canadian workforce was women in 2018. Since diversity is growing and becoming increasingly prevalent in workplaces across Canada, employers are required to adapt to this diversity quickly. To meet this need, Electricity Human Resources Canada has developed resources to support the inclusion of underrepresented groups such as women, indigenous, immigrants, and individuals with disability in the electricity sector. The majority of participants in this study were of white European/American ancestry (89.7%), males (95.0%) and mostly born and raised in Ontario (87.0%). The male and Caucasian dominance of the employers in this sample (that formed the majority of our sample) may explain the low diversity in this study. Future studies may consider a larger sample size with a more diverse group of participants and perform an intersectional analysis using gender-based analysis, plus incorporating minority identities in the analyses.

Further to our quantitative findings, our qualitative findings (to be published elsewhere) emphasized the continued leverage of engagement and support of employers in the construction trades as an important avenue for growth to address the ongoing shortage of skilled trades workers in Ontario, Canada. Our current study strengths include the uniqueness of the setting and the paucity of prior research with this population. Although several studies have investigated burnout in employees, there is a paucity of studies examining the level of burnout in employers, specifically in small businesses where the employers have similar situations and activities with their employees (e.g., long hours of working, fewer holidays, and breaks, isolation in the work environment, etc.). Therefore, future studies need to evaluate mental health in this population, especially due to the impact that the COVID-19 pandemic has had on small businesses.

The small sample size was one of the main limitations of this study. Another limitation is the lack of validity and reliability of CBI in electricians and plumbers that was used to assess burnout in our study participants. In recent years, an increasing number of studies have used the CBI [34]. The CBI has been tested for validity and reliability among physicians [36,37,38], pharmacists [39], nurses [35,38], medical students [50], other healthcare employees [38,51,52], and teachers and professors [53,54,55]. However, no psychometric studies of the CBI in electrician and plumber populations exist. Therefore, an examination of the psychometric properties of the CBI in these populations is needed.

## 5. Conclusions

This study’s findings represent the lack of diversity at the employer level in the electrical and plumbing trades. This disparity may reinforce the difficulty in recruitment and retention through themes identified in our qualitative analysis and the lack of high burnout observed, given the homogenous sample in this study. Future large studies should look to clarify the role of diversity in improving mental health awareness and trade stigma.

## Figures and Tables

**Table 1:** Demographic characteristics of study participants (*n* = 40).

Demographic Variables			
		Mean	SD
Age		47.97	15.62
		Frequency (*n*)	Percent (%)
Born and/or raised in Ontario	Yes	35	87.5
	No	5	12.5
Marital Status	Married/Common-law	32	80.0
	Single	6	15.0
	Divorced	1	2.5
	Widowed	1	2.5
Education Level	Completed high school	11	27.5
	College certificate/diploma	22	55.0
	University Undergraduate and graduate	4	10.0
	Other	3	7.5
Training obtained in Ontario	Yes	39	97.5
	No	1	2.5
Primary language	English	36	90.0
	French	1	2.5
	Spanish	1	2.5
	Ukrainian	1	2.5
	Romanian	1	2.5
Ethnicity	White North American	29	74.3
	White European	6	15.4
	Other	2	5.1
	Asian East	1	2.6
	Aboriginal	1	2.6
Smoking	Non-smoker	27	67.5
	smoker	13	32.5
Current employment	Employed in electrical sector	37	92.5
	Employed in plumbing sector	2	5.0
	Employed in non-electrical/plumbing sector	1	2.5
Belong to a Union	No	37	92.5
	Yes	3	7.5
Average overtime hours per week	None	21	60.0
	5 h or less	5	14.3
	6–10 h	7	20.0
	More than 10 h	2	5.7
Intend to stay in current position for the next 5 years *	Yes	31	94.0
	No	2	6.0
Gross annual income	Less than $80,000	13	32.5
	$80,000 or more	19	47.5
	Prefer not to answer	8	20.0

* One participant retired in 2019, and 5 participants will be retired within the next 5 years.

**Table 2:** Copenhagen Burnout Inventory (*n* = 40).

Type of Burnout	Mean [SD]	Minimum Score	Maximum Score	Median	Moderate Burnout (*n*)	High Burnout (*n*)	Severe Burnout (*n*)
Personal burnout	33.48 [16.60]	4.16	70.83	33.33	7	0	0
Work-related burnout	25.63 [13.21]	7.14	67.85	21.42	2	0	0
Colleague-related burnout	17.56 [16.32]	0.00	54.16	16.66	2	0	0

## Data Availability

The data presented in this study are available on request from the corresponding author. The data are not publicly available due to privacy.

## Supplementary Materials

The following supporting information can be downloaded at: https://www.mdpi.com/article/10.3390/bs12120505/s1, Table S1: Association between the availability of work-related factors to the satisfaction of the participants and average burnout (*n* = 40); Table S2: Association between the importance of work-related factors and burnout in the study participants (*n* = 40). Additional information can be provided upon request from the corresponding author ((B.N.-K.).
