# Supplementary material for: Mental Health Outcomes among Electricians and Plumbers in Ontario, Canada: Analysis of Burnout and Work-Related Factors
Source: Behav Sci (Basel). 2022 Dec 12;12(12):505. doi: 10.3390/bs12120505 (PMC9774700; doi:10.3390/bs12120505)
Supplement: Supplementary file 1 [file behavsci-12-00505-s001.zip › behavsci-2059959-supplementary.pdf]

Table S1. Association between availability of work-related factors to satisfaction of the participants and average burnout (n=40)

**Factors**	**Satisfied with availability of work-related factors (%)**	**SE of percent of satisfied**	**Personal burnout**				**Work-related burnout**				**Colleague-related burnout**			
			Mean difference*	SE difference	Z value**	P value	Mean difference	SE difference	Z value	P value	Mean difference	SE	Z value	P value
Career advancement possibility	68	7.3	2.70	5.91	-0.35	0.72	3.26	4.78	-0.27	0.78	1.55	5.85	-0.32	0.74
Effective management	69	7.3	-1.22	7.03	-0.10	0.91	0.73	4.98	-0.05	0.95	5.03	6.06	-0.97	0.33
Facility and workplace equipment/resources	62	7.6	-5.62	5.72	-1.04	0.29	-1.55	4.63	-0.58	0.55	1.65	5.65	-0.36	0.71
Financial support for external training	51	7.9	-2.01	5.75	-0.59	0.55	7.07	4.46	-1.01	0.30	15.63	5.11	-2.73	0.006
Flexible scheduling for external training	72	7.0	3.24	6.22	-0.23	0.81	9.49	4.76	-1.16	0.24	10.26	6.04	-1.51	0.12
Flexible scheduling for family commitments	75	6.8	-0.12	6.46	-0.12	0.89	2.46	5.19	-0.14	0.88	-0.21	6.51	-0.09	0.92
Full-time employment opportunity	86	5.4	-2.40	8.08	-0.41	0.67	4.50	6.47	-0.09	0.92	-3.19	8.13	-0.48	0.62
Internal staff development opportunity	58	7.8	0.82	5.67	-0.06	0.94	3.33	4.54	-0.87	0.38	4.51	5.66	-1.15	0.25
Involvement in organization decision-making	72	7.0	2.08	6.24	-0.44	0.65	1.58	5.03	-0.37	0.70	-0.12	6.29	-0.16	0.87
Leave of absence for external training	64	7.5	-2.96	5.80	-0.54	0.58	1.48	4.69	-0.33	0.73	4.51	5.82	-1.06	0.28
Orientation program for new staff	53	7.8	0.17	5.61	-0.01	0.98	6.16	4.39	-1.23	0.21	7.22	5.51	-1.29	0.19
Peer support	56	7.8	0.72	5.63	-0.06	0.94	4.30	4.48	-0.45	0.65	1.51	5.67	-0.49	0.61
Salary and benefits	60	7.7	2.30	5.81	-0.15	0.87	5.23	4.61	-0.64	0.51	5.07	5.42	-0.85	0.39
Stable staffing/minimal turnover	58	7.8	3.20	5.65	-0.46	0.64	6.11	4.45	-0.97	0.33	10.23	5.44	-1.80	0.07
Workload allocation	61	7.7	4.82	5.68	-0.58	0.55	5.51	4.53	-0.75	0.45	7.18	5.65	-1.34	0.17
Workplace safety	71	7.1	6.91	5.88	-0.91	0.35	7.16	4.90	-0.83	0.40	6.10	6.25	-1.12	0.26
Opportunity to become a fully licensed electrician (For apprentices)	81	6.2	-4.41	7.36	-0.40	0.68	5.70	6.21	-0.30	0.76	3.37	7.55	-0.20	0.83
Opportunity/support to qualify as Master Electrician	69	7.3	4.10	6.06	-0.51	0.60	9.19	4.93	-0.98	0.32	3.23	6.28	-0.57	0.56

*Difference in average burnout score between those who were unsatisfied with the availability of the factors and those who were satisfied with. Positive values indicate that unsatisfied participants with availability of the factors had higher level of burnout.

**Mann–Whitney U test.

Table S2. Association between the importance of work-related factors and burnout in the study participants (n=40)

**Factors**	**Recognized as important factor (%)**	**SE of recognized important factors (%)**	**Personal burnout**				**Work-related burnout**				**Colleague-related burnout**			
			Mean difference	SE difference	Z value	P value	Mean difference	SE difference	Z value	P value	Mean difference	SE difference	Z value	P value
Career advancement possibility	59	8.2	11.96	5.18	-2.25	0.02	11.55	4.02	-2.45	**0.01**	9.25	5.45	-1.59	0.11
Cost of Living	84	6.1	2.22	7.40	-0.35	0.72	6.67	5.84	-0.52	0.60	-4.87	7.51	-0.93	0.48
Existing leadership	73	7.4	4.69	6.10	-0.77	0.43	8.09	4.74	-1.65	0.09	0.87	6.27	-0.19	0.84
Facility and workplace equipment/resources	70	7.6	0.05	6.04	-0.15	0.88	4.74	4.73	-1.87	0.06	0.75	6.09	-0.20	0.84
Family commitments	92	4.5	-14.14	9.72	-1.28	0.19	-10.10	7.85	-1.60	0.10	-18.79	9.69	-2.28	0.02
Financial support for external training	57	8.3	-4.54	5.46	-1.11	0.26	3.51	4.39	-0.68	0.49	-0.15	5.62	-0.38	0.69
Flexible scheduling for external training	69	8.0	0.44	6.17	-0.07	0.94	7.47	4.76	-1.65	0.09	2.75	6.26	-0.07	0.94
Full-time employment opportunity	76	7.2	6.32	6.28	-0.97	0.32	7.23	4.96	-1.46	0.14	1.03	6.49	-0.26	0.78
Income and benefits	95	3.8	-13.73	11.85	-0.87	0.38	-8.55	9.59	-0.98	0.32	-18.26	11.92	-1.83	0.06
Internal staff development opportunity	68	7.8	1.01	5.83	-0.44	0.66	3.08	4.65	-1.27	0.20	-4.49	5.90	-0.85	0.39
Involvement in organization decision-making	83	6.0	-6.38	7.43	-0.66	0.50	-2.12	6.01	-0.02	0.98	-4.63	7.52	-0.98	0.32
Leave of absence for external training	49	8.3	-3.54	5.43	-0.62	0.53	-0.72	4.38	-0.59	0.55	-8.01	5.40	-1.61	0.10
Orientation program for new staff	68	7.8	0.50	5.84	-0.29	0.76	6.07	4.57	-1.35	0.17	-3.46	5.92	-0.93	0.34
Peer support	73	7.4	-3.30	6.13	-0.48	0.63	1.72	4.93	-0.74	0.45	4.30	6.22	-0.41	0.67
Stable staffing/minimal turnover	86	5.7	-8.85	7.85	-0.98	0.32	-1.92	6.41	-0.24	0.80	-2.63	8.13	-0.85	0.39

Workload allocation	81	6.5	-3.25	6.95	-0.13	0.89	-3.06	5.80	-0.25	0.79	-6.62	7.02	-1.37	0.16
Workplace Safety	86	5.7	-7.31	7.90	-0.73	0.46	-4.40	6.37	-0.67	0.50	-6.48	8.07	-1.17	0.24
Opportunity to become a fully licensed Electrician	65	8.7	0.68	6.16	-0.14	0.88	8.54	6.11	-0.85	0.39	7.54	6.10	-0.94	0.34
Opportunity/support to qualify as Master Electrician	61	8.9	3.94	5.91	-0.50	0.61	9.60	4.71	-1.22	0.21	8.00	5.90	-1.08	0.27
